# Preparation and Characterization of Poly(vinyl-alcohol)/Chitosan Polymer Blend Films Chemically Crosslinked with Glutaraldehyde: Mechanical and Thermal Investigations

**DOI:** 10.3390/molecules29245914

**Published:** 2024-12-14

**Authors:** Daniel Pugar, Tatjana Haramina, Mirela Leskovac, Lidija Ćurković

**Affiliations:** 1 Department of Polytechnic, Dr. Franjo Tuđman Defense and Security University, Ilica 256b, 10000 Zagreb, Croatia; 2 Faculty of Mechanical Engineering and Naval Architecture, University of Zagreb, I. Lučića 5, 10000 Zagreb, Croatia; lidija.curkovic@fsb.unizg.hr; 3 Department of Surface Engineering of Polymer Materials, Faculty of Chemical Engineering and Technology, University of Zagreb, Marulićev trg 19, 10000 Zagreb, Croatia; mlesko@fkit.unizg.hr

**Keywords:** polymer blends, polymer nanocomposites, poly(vinyl-alcohol), chitosan, dynamic mechanical analysis, barrier properties, time-lag method

## Abstract

In this study, poly(vinyl-alcohol) (PVA)/chitosan (CS) polymer blend films with different amounts of CS (0, 5, 20 and 35 wt. %) crosslinked by glutaraldehyde (GA) were prepared. The structure and properties of the prepared polymer films were studied by means of dynamic mechanical analysis (DMA), differential scanning calorimetry (DSC), and the time-lag permeation technique. The DMA analysis showed that CS reduces the crystallinity degree of PVA, leading to a higher amount of the amorphous phase contributing to the *α* relaxation that corresponds to the glass-to-rubber transition. However, the mobility of the amorphous phase can be restricted with crosslinking with 1 wt. % GA. Interaction between the PVA and the CS was confirmed by DCS analysis. Additionally, the influence of the CS and crosslinking on the permeation of nitrogen molecules was investigated. The permeation was examined by the time-lag method. It was found that the addition of CS and GA to PVA improves barrier properties.

## 1. Introduction

Non-toxic lightweight materials have growing applications in various fields of everyday life, especially in medicine, pharmacy, and the food industry. The blending of polymers is one way of improving materials or creating new materials with desired specific properties. It is a complex discipline since polymers are thermodynamically conditioned, not inclined to mixing. That is because polymers are intrinsically disordered systems and by mixing them, entropy will not increase significantly. The miscibility of two different polymers is dependent on the specific interactions between polymer chains and is possible only when the different polymers can establish specific interactions between their chains, causing negative enthalpy and Gibbs free energy of mixing [1].

The mixing of different polymers in solutions is one way to achieve a blend at the molecular level. The resulting properties depend on the properties of the input polymers. Studies with the aim to optimize blend properties attract great attention, as the costs of blending existing materials are significantly lower than the costs of synthesizing and testing a completely new polymer. Whether the blend will have properties between the input components, or will be significantly different, depends on their miscibility [1].

Poly(vinyl-alcohol) (PVA) is a synthetic polymer, colourless and odourless. It is prepared by hydrolysis of poly(vinyl-acetate) (PVAc), where the acetate groups are replaced by hydroxyls [2,3]. The molecular weight of PVA varies between 20 and 200 kDa [4,5]. PVA is a non-toxic [6,7], water-soluble [8], biodegradable, and biocompatible polymer [9]. It is characterized by high flexibility and good barrier properties to oxygen and carbon dioxide [10,11]. These properties make it very suitable for application in medicine and pharmacy, as well as in the food, textile, and cosmetic industries [2,5,12,13,14,15]. The proportion of crystal phase and properties of this polymer depend on molecular weight, tacticity, and degree of hydrolysis. The crystallized PVA is a stable and chemically inert polymer [5]. The degree of crystallinity can be increased by repeated freezing cycles at −20 °C and thawing at room temperature [3,12]. The properties of PVA can also be enhanced with chemical crosslinking, which significantly influences its physical characteristics. The level of modification is influenced by both the degree of crosslinking and the degree of crystallinity. Among the various crosslinking methods, the aldehyde-crosslinking approach is the most widely used, due to high crosslinking efficiency [16,17]. Glutaraldehyde (GA) is a commonly used crosslinking agent for PVA [18,19], and was chosen for use in this study. It is well established that aldehyde groups (-CHO) of glutaraldehyde can react with the hydroxyl groups (-OH) of the PVA polymer chain, resulting in the formation of a dense three-dimensional network structure [20,21]. This modification can further improve the mechanical properties and stability of PVA, making it even more suitable for specialized applications such as wound dressing, artificial organ preparation, contact lenses, and drug delivery systems. PVA can also be mixed with other polymers and organic nanoparticles [12].

CS is a linear copolymer composed of randomly distributed β-(1-4)-linked D-glucosamine (deacetylated unit) and N-acetyl glucosamine (acetylated unit) [12,22,23]. It is the second most abundant natural polysaccharide, next to cellulose, obtained by alkaline deacetylation of chitin, which is the major structural component of exoskeletons and crustaceans such as crabs, shells, lobsters, and shrimps [24]. CS has excellent properties like non-toxicity, biodegradability, biocompatibility, and antimicrobial activity [25,26,27]. It has a wide range of applications in food [28], in the medical and pharmaceutical industries, and in biomedicine and agriculture [1,27,29]. The main disadvantage of CS is fragility, and it can be reduced by mixing with flexible PVA.

CS contains hydroxyl and amine groups and is potentially miscible with PVA [30,31]. The contribution to the negative enthalpy of mixing results from the strong hydrogen bonds between them [32]. Due to the specific intermolecular interactions, the blend of PVA/CS has good mechanical properties [33]. Therefore, CS was blended with PVA matrix to investigate the thermal behaviour of PVA matrix in presence of CS at various proportions. The miscibility of polymers is most often analysed by measuring the glass transition temperature (*T_g_*) and by comparing it with the *T_g_* of the constituents. When PVA and CS are mixed in a solution, it is observed that the viscosity of the solution increases with increasing concentration of chitosan [34]. Also, the increase in chitosan concentration reduces the crystallinity of PVA [1,35].

The properties of the PVA/CS blend can also be modified through chemical crosslinking with glutaraldehyde, a commonly used crosslinking agent for PVA/CS blends due to its availability and low cost [36,37,38,39]. The crosslinking of chitosan with glutaraldehyde occurs through a reaction between the amino groups (-NH_2_) of the chitosan polymer chain and the carbonyl (aldehyde) groups (-CHO) of glutaraldehyde [40]. This reaction is based on the Schiff base mechanism [27]. The aldehyde groups of glutaraldehyde easily react with the amino groups of chitosan, forming imine bonds (Schiff base (C=N)), and with the hydroxyl groups of PVA, forming acetal bonds, thereby enhancing the effectiveness of glutaraldehyde in the simultaneous crosslinking of the PVA/chitosan blend [41].

Application of the described polymer materials is determined by a wide range of their properties. In addition to their antimicrobial activity, it is necessary to learn and optimize their mechanical and thermal properties, as well as gas permeability. The mechanical properties, energy absorption, and *T_g_* of flexible polymers, such as PVA, are highly dependent on the time scale of the experiment (e.g., the deflection rate) and temperature [15]. If the load is dynamic, the time scale is determined by the frequency. The capability of the material to damp out mechanical vibrations is called internal friction. For polymers, it is a consequence of macromolecular dynamics. Dynamics of molecules, segments, side groups, or vibrations in the backbone that contribute the internal friction can be studied by means of DMA. From relaxation processes visible in the mechanical spectra in combination with DSC analysis, information on structure and mechanical properties depending on temperature can be obtained. In the application typical for these materials, the permeation of small gas molecules such as O_2_, N_2_, or CO_2_ is very important. The addition of CS reduces the crystalline phase proportion of PVA, leading to more free volume needed for molecules to permeate through the material. In general, a less ordered structure, i.e., lower density, results in the higher permeability of small molecules soluble in the polymer. Therefore, in this study, poly(vinyl-alcohol) (PVA)/chitosan (CS) polymer blend films with different amounts of CS crosslinked by glutaraldehyde (GA) were prepared, and their structure and properties were investigated.

## 2. Results and Discussion

### 2.1. Dynamic Mechanical Analysis

**Figure 1:**
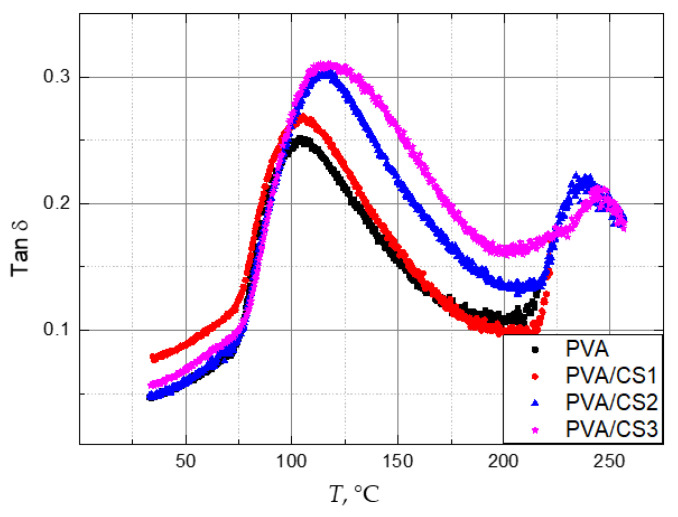
Temperature dependence of tan *δ* for PVA films with 0 wt. % (PVA), wt. % (CS1), 20 wt. % (CS2), and 35 wt. % (CS3) of CS.

Dynamic mechanical analysis (DMA) is the most sensitive known method for observing changes in material structure [42]. It measures the damping of mechanical vibrations due to a phase shift δ between stress and strain over a specified temperature range during constant heating and/or cooling rate, and the corresponding complex modulus E* = *E*′ + *iE*″ [43]. DMA spectra of PVA films with 0, 5, 20, and 35 wt. % of CS are represented in Figure 1. The damping factor tan *δ* is a quantity related to the energy absorption caused by changes in the molecule’s conformation. In this study, only the α relaxation attributed to the glass-to-rubber transition was analysed, and less pronounced relaxation processes in the low temperature range attributed to movements on a smaller scale (movement of side groups, vibrations of covalent bonds, etc.) were not considered. In the glassy state, the damping is low because macromolecules do not change configuration during a short cyclic load, but deformations occur due to local atomic motions. By heating, the macromolecules begin to move, and the damping increases. When the maximum tan *δ* is reached, the mobility of the macromolecules is already sufficiently facilitated, resistance to movements is reduced, and there is a drop in internal friction which is manifested in the fall of the damping factor tan *δ*. *T_g_* was determined as the onset temperature of the α relaxation. In addition to that, it should be borne in mind that one relaxation peak is the sum of different molecule responses to mechanical excitation, hence structural changes towards inhomogeneity are seen as peak broadening.

The α relaxation of pure PVA begins at a temperature of 75 °C and reaches its maximum at 103.8 °C. With the addition of CS, the onset of the relaxation process slightly shifts to higher temperatures, indicating that CS reduces the flexibility of PVA. Due to intra- and intermolecular forces, CS has a very rigid structure. Its poor mechanical properties (high rigidity and fragility) are most often cited as its main drawback [22,44,45]. By adding 5, 20, and 35 wt. % of CS, the peak broadens and consequently *T_g_* increases by 1.5 °C, 10.8 °C, and 13.4 °C, respectively. The onset, maximum, and end temperatures of α relaxation for PVA/CS blends with different compositions are shown in Table 1.

In this study, it was not possible to analyze pure CS, due to its brittleness. From the mechanical spectra presented in [46], its onset of the α relaxation process visible in the tan *δ* diagram is at about 150 °C. The contribution of CS to the observed peak is visible as the shift of the maximum and the broadening. Additionally, CS reduces the crystallinity degree of PVA [47,48,49], leading to a higher amount of the amorphous phase, contributing to the intensity of α relaxation (tan *δ*_max_). The values of tan *δ*_max_ for PVA/CS blends with different compositions are shown in Table 1. The difference in the α relaxation intensity of films with 5 and 20 wt. % CS was 11.8%, and this is the largest increase. A further addition of CS to 35% caused an increase of only 1.6%.

**Figure 2:**
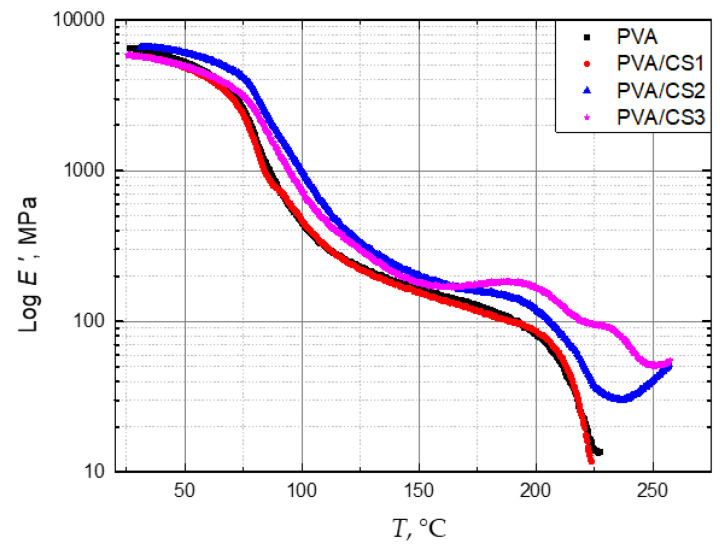
Storage modulus *E*′ vs. temperature for PVA films with 0 wt. % (PVA), wt. % (CS1), 20 wt. % (CS2), and 35 wt. % (CS3) of CS.

Figure 2 shows the temperature dependence of the storage module *E*′ of PVA/CS blends with different compositions. In the glassy state, the *E*′ of all the materials is high. Due to the cyclic motion, with increasing temperature, there is a pronounced drop in *E*′, by several orders of magnitude, which corresponds to the glass transition of the polymeric material.

At room temperature, where the blends are in the glassy state, the *E*′ of PVA/CS1 and PVA/CS3 was 8.6% and 9.8% lower than that of pure PVA, respectively (about 600 MPa), while the *E*′ of the PVA/CS2 blend was 3.6% higher (150 MPa). Thus, the addition of 20 wt. % of CS increases the stiffness of PVA in the glassy state, while at a mass fraction of 35 wt. %, the stiffness in the glassy state decreases. This can be explained by the fact that CS molecules disrupt the compact structure of PVA, i.e., as the proportion of CS in the blend increases, the structure becomes rougher, and areas of incompatibility appear [50]. In Figure 2, a drastic reduction in *E*′, starting at about 75 °C, is attributed to the α relaxation. The addition of CS increases *E*′ at temperatures above *T_g_*, which indicates an increase in the stiffness of the material in the rubbery state. Moreover, above *T_g_*, the decay of *E*′ in blends with 20 and 35 wt. % CS is slower than that of neat PVA and PVA with only 5 wt. % CS. An overview of *E*′ at characteristic temperatures, i.e., room temperature (25 °C), the onset of the α relaxation (75 °C), and the α relaxation peak (110 °C), is given in Table 2.

In the rubbery state, all PVA/CS blends have higher storage moduli *E*′ than in pure PVA (Table 2). The PVA/CS2 blend shows the highest *E*′ of all the tested materials in the entire temperature range, both in the glassy and in the rubbery state. Near *T_g_* (at about 75 °C), the *E*′ of the PVA/CS2 blend is higher by 65%.

In other studies, the ratio of 80:20 of PVA and CS is stated as the most favorable in terms of mechanical properties [47,51]. At this ratio, the films are strong enough, and still have a good flexibility. The further addition of CS leads to areas of incompatibility, as evidenced by numerous SEM analysis of PVA/CS blends.

Crosslinking has a great influence on the dynamic mechanical properties of materials, especially near the α relaxation. In general, materials with a higher degree of crosslinking have a broader α relaxation peak with a lower intensity (attenuation intensity is reduced) shifted toward higher temperatures and consequently less effective reduction in *E*′ with temperature growth [52].

**Figure 3:**
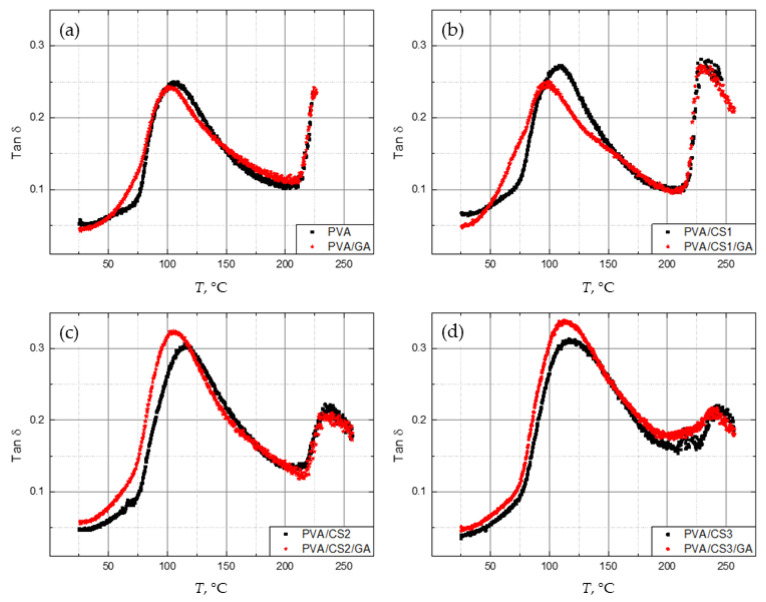
Damping factor tan *δ* as a function of temperature for crosslinked and non-crosslinked PVA films with the following mass fractions of CS: (**a**) 0 wt. %, (**b**) 5 wt. %, (**c**) 20 wt. %, (**d**) 35 wt. %.

By crosslinking, two competitive effects occur in the structure of material. On the one hand, the amount of amorphous phase that contributes to α relaxation increases. Likewise, the amount of free volume in the structure increases, resulting in a reduction in material rigidity. On the other hand, with the addition of a crosslinking agent, mobility within the amorphous phase is hindered due to crosslinking. Consequently, depending on the amount of crosslinking agent and the proportion of amorphous phase in the material, the crosslinking may have different effects on the relaxation intensity. High density of crosslinks reduces flexibility, whereas low density of crosslinks can even increase it. Figure 3 shows the DMA spectra of the damping factor tan *δ* depending on temperature, for all the crosslinked PVA/CS films together.

After crosslinking, the glass-to-rubber transition in all films takes place at a lower temperature range, which means that the flexibility of the macromolecules was increased. The higher amount of free volume reduces the energy necessary for the onset of the α relaxation.

In general, in most cases the *T_g_* increases with the crosslinking degree, because the crosslinking increases polymer rigidity. The hydroxyl groups in PVA contribute to stiffness via hydrogen bonding. When the number of hydroxyl groups is reduced, either by crosslinking or by branching due to the grafting of the crosslinker, the hydrogen bonding is attenuated, and the stiffness is reduced. Hence, the crosslinker induces less stiffness than the hydrogen bonding [53]. With the crosslinker addition, the strong hydrogen bonds are replaced with crosslinks. The consequent reduction in the crystallinity degree increases the amount of free volume. As a result, the crosslinked PVA can have a more flexible structure, and hence, a lower *T_g_* than the unmodified PVA.

There is no significant difference in the α relaxation peak height between the crosslinked and non-crosslinked PVA, whereas the onset temperature is much lower for the crosslinked PVA film.

As the amount of CS increases, the temperature difference between the onset and the maximum of α relaxation between crosslinked and non-crosslinked blends reduces. The crosslinker GA has a greater impact on the materials that are more inclined to crystallization before crosslinking (films with more PVA). The blend with only 5 wt. % CS (PVA/CS1) still has a high crystallinity degree. By adding GA, the amount of free volume is increased, which contributes to flexibility. For both PVA and PVA/CS1 blends, the onset is changed compared to non-crosslinked blends, which means that segments participating in relaxation processes at lower temperatures are affected by structural changes. With the addition of GA, grafting of crosslinker molecules occurred and the dynamics of new branches contributed to the relaxation at lower temperatures.

As the ratio of CS increases, the structure is less ordered and the amount of free volume in the structure increases. Due to the high amount of amorphous phase and free volume prior to crosslinking, blends with 20 and 35 wt. % show only a slight increase in flexibility with crosslinking, especially at the beginning of relaxation. In the DMA spectra, the least differences in peak temperature between crosslinked and non-crosslinked films occur in the pure PVA and the PVA/CS blend with the highest amount of CS. The temperatures of the onsets and the maxima of α relaxation for all the films are shown in Table 3.

Crystallinity degree and free volume affect the intensity of the α relaxation. With the addition of GA, the intensity of the films with higher crystallinity degrees decreases, i.e., PVA and PVA/CS1, while that of the PVA/CS2 and PVA/CS3 blends increases (Table 4). PVA, CS, and GA molecules have the ability to penetrate and intertwine with each other, forming interpenetrating networks [36,54]. This intertwining of molecules greatly disrupts their proper arrangement, reducing the crystallinity of both PVA and CS. Therefore, the addition of crosslinker GA further increases the amount of amorphous phase in the structure, contributing to α relaxation. Disordered structure and crosslinks have the opposite effect on the intensity. The addition of the small amount of crosslinker GA is more effective in films with less CS.

**Figure 4:**
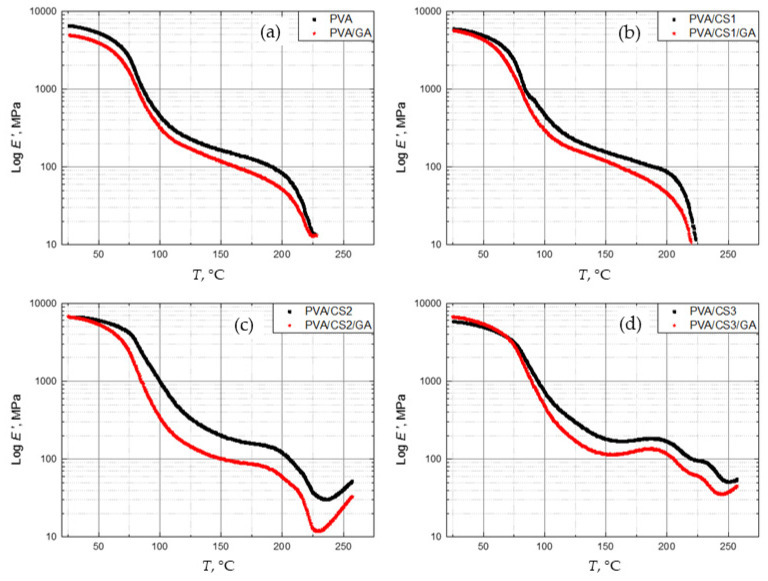
Storage modulus *E*′ as a function of temperature of crosslinked and non-crosslinked PVA films with different amounts of CS: (**a**) 0 wt. %, (**b**) 5 wt. %, (**c**) 20 wt. %, (**d**) 35 wt. %.

Figure 4 shows the DMA spectra of *E*′ vs. temperature for all films. The crosslinked blends have lower *E*′, which is consistent with the results of the damping factor measurements. The drop in *E*′ proves that the small amount of crosslinker GA increases the flexibility of the PVA/CS films.

However, prior to the glass transition, the stiffness or *E*′ of crosslinked films increases with CS content. The crosslinking of pure PVA film in the glassy state reduced the *E*′ by 25%. The same effect was visible for the blend with only 5 wt. % CS with a smaller difference of 4.7%. At room temperature, the crosslinked blends with 20 and 35 wt. % CS have a higher *E*′, up to 30 °C and 75 °C, respectively, when its decrease is more pronounced than in other blends and the films become more flexible. The higher *E*′ of crosslinked blends with more CS at low temperatures, i.e., in the glassy state, coincide with the high brittleness of the prepared bends. In the rubbery state, all crosslinked blends have a lower *E*′ and a higher flexibility than non-crosslinked films.

**Figure 5:**
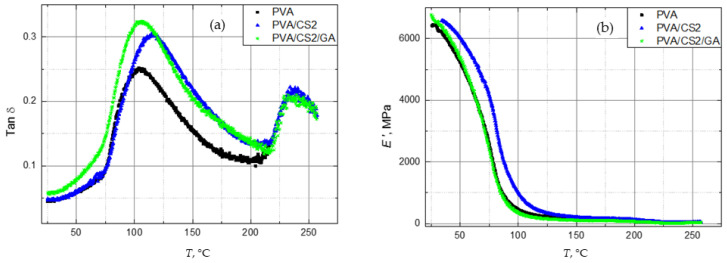
DMA spectra of pure PVA, PVA with 20 wt. % of CS (CS2) and crosslinked PVA with 20 wt. % of CS (CS2/GA); (**a**) damping factor tan *δ* and (**b**) storage modulus *E*′ depending on temperature.

Previous studies [47,51] indicate that the PVA/CS blend with 20 wt. % CS has the most favorable mechanical properties, high strength, and satisfactory flexibility. In Figure 5, tan *δ* and *E*′ of these blends, both neat and crosslinked, are compared to the neat PVA.

The addition of CS leads to a reduction in the flexibility of PVA, but this can be improved with crosslinking. In the tan *δ* spectrum (Figure 5a), CS shifts the PVA *T_g_* towards higher values and increases rigidity. The crosslinker GA in PVA/CS blends returns the *T_g_* to lower values and increases their flexibility. CS increases the intensity of α relaxation due to the higher amount of the amorphous phase in the structure, but mobility of the amorphous phase can be reduced with crosslinking. In blends with low CS content and thus a low amorphous phase content, the mobility of the amorphous phase can be reduced by the addition of 1 wt. % of GA. In blends with a higher CS content, this amount of crosslinking agent is not enough to reduce the mobility of a large amount of amorphous phase. On the contrary, in such blends, the crosslinker further increases the amount of free volume, and the density of crosslinking is not enough to prevent its mobility, resulting in a higher intensity of relaxation (Figure 5a). In the DMA spectrum showing the temperature-dependent storage modulus *E*′, it is clearly seen that chitosan increases the stiffness of PVA, but it can be reduced with crosslinking (Figure 5b). The addition of crosslinker GA returns the modulus of PVA/CS films to values corresponding to the modulus of the neat PVA film.

### 2.2. Results of Differential Scanning Calorimetry (DSC)

**Figure 6:**
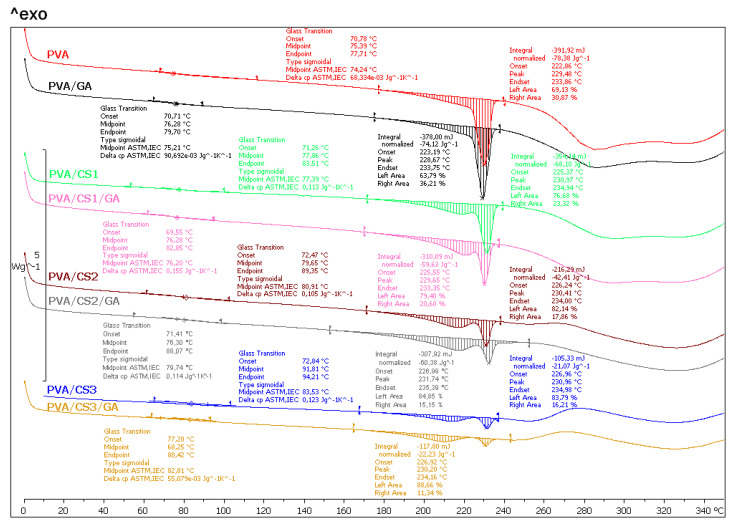
DSC thermograms of PVA/CS blends crosslinked with glutaraldehyde and with different amounts of CS: 0 wt. % (PVA), 5 wt. % (CS1), 20 wt. % (CS2), and 35 wt. % (CS3).

Figure 6 shows the thermograms of the second heating cycle of neat PVA and PVA/CS blends. The DSC curve of neat PVA shows a glass transition at 74.2 °C and an endothermic melting transition with a maximum at 229.5 °C. The area below the endothermic peak represents the melting enthalpy, which is 78.4 J g^−1^ for the neat PVA matrix. The neat CS had no endothermic peak, which indicates that the structure is disordered [55].

The addition of the crosslinker GA led to a slight change in the glass transition and melting temperature, while the glass transition shifted to higher temperatures when the CS content was increased, indicating a decrease in the flexibility of PVA due to the interactions between PVA and CS. The glass transition temperature of the blends shifted by 3.2 °C (CS1), 6.7 °C (CS2), and 9.3 °C (CS3) compared to pure PVA.

In order to analyze the influence of the CS and GA on the crystallization of the PVA matrix, the melting temperature *T_m_* and the melting enthalpy Δ*H_m_* of PVA/CS and PVA/CS/GA blends were analysed. The analysis of the melting temperature showed a negligible change in the melting temperature of the blends compared to the neat PVA.

The comparison curves of the second heating cycle show that the unimodal melting curve changes to a bimodal one with increasing CS content and decreasing melting enthalpy, indicating the destruction of the crystal structure and the formation of less ordered crystal forms that melt at a lower temperature in the temperature range of about 180 °C to 220 °C. The main melting peak at about 229 °C does not change significantly with an increase in the amount of CS. The slightly higher melt enthalpy value with the addition of GS crosslinker indicates the presence of GS interactions with PVA and CS.

**Figure 7:**
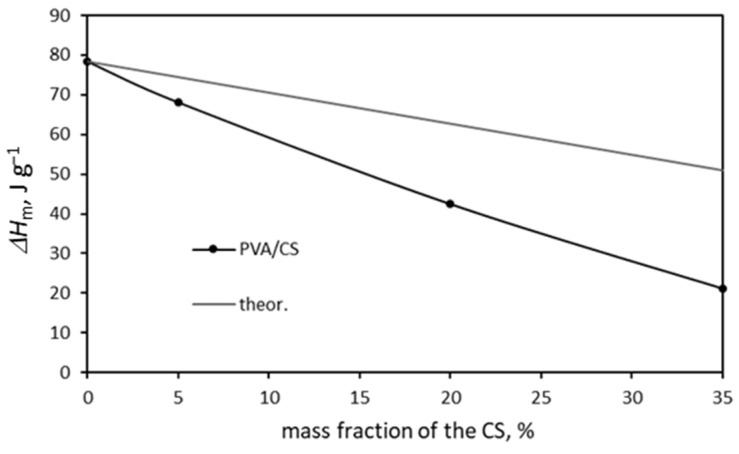
Melting enthalpy of PVA/CS blends.

The values of the melting enthalpy as a function of the mass fraction of CS are shown in Figure 7. All the values of the melting enthalpy of the PVA/CS mixtures are below the theoretical values, which confirms that the CS hinders and interferes with the crystallization of the PVA matrix.

In conclusion, blending the PVA induced morphological and chemical modifications. The former involves the thickness of the crystallites and the degree of crystallinity, and the latter are caused by the crosslinking and by the branching due to the grafting of the GA crosslinker [56]. Both effects were related to each other. The endothermic peak decreased rapidly, both for samples with and without added GA, but it was still visible for 35 wt. % of CS.

DSC analysis confirmed the results of the DMA analysis. It was confirmed that CS shifts the glass transition temperature of PVA to higher temperatures and reduces its degree of crystallinity. Although with an increase in the proportion of CS, the melting point of PVA does not shift to lower values, a decrease in the enthalpy of melting is seen, which proves that CS hinders and disturbs the crystallinity of PVA.

### 2.3. Permeability of Nitrogen

**Figure 8:**
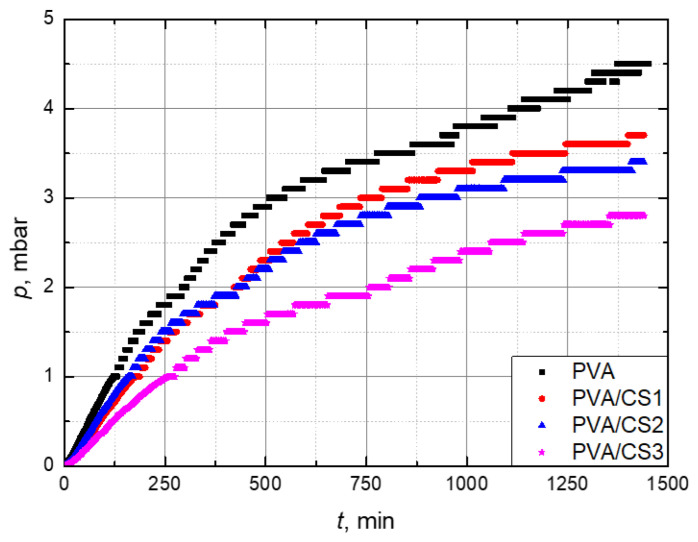
Results of permeability measurements for PVA films with different amounts of CS: 0 wt. % (PVA), 5 wt. % (CS1), 20 wt. % (CS2), and 35 wt. % (CS3).

For each type of material, three measurements were performed to obtain good reproducibility. It is important to note that at the beginning of the measurement, there is no visible delay area (the so-called time lag) where the gas does not pass currently through the film, but first adsorption of gas from the high-pressure system occurs, then sorption and finally desorption on the low-pressure side of the system. Therefore, only a stationary area was observed where linear pressure growth was realized. The permeation results are displayed graphically, and permeability was calculated from that graph. Figure 8 shows the results of permeability measurement for films of each type of PVA/CS blend. From the results, it is apparent that the linear area comes after about 500 min from the beginning of the test; therefore, for further analysis, only that area was observed.

**Figure 9:**
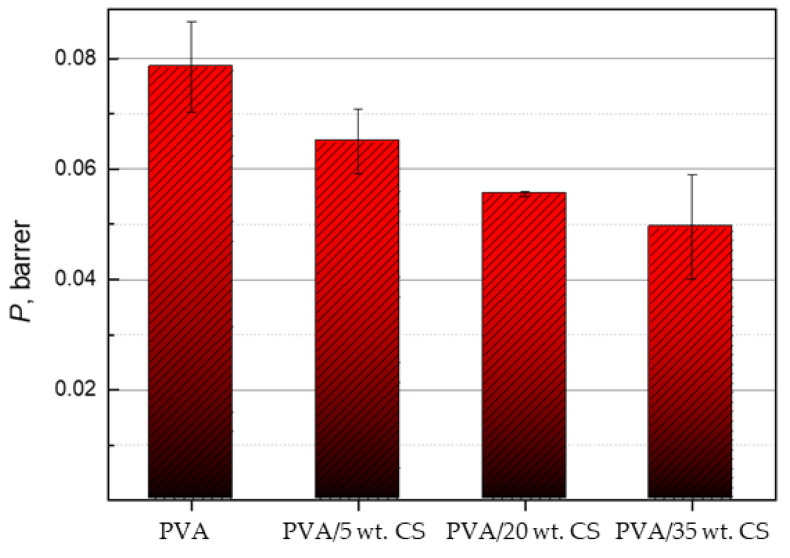
Influence of CS on the permeability of PVA films; data are representative of the results of repeated experiments (n = 3) and expressed as the mean value.

According to the calculated values of permeability, it can be concluded that the addition of CS improves the barrier properties of PVA films on nitrogen. Since there are no available data for nitrogen permeation through PVA/CS films, the results are compared with oxygen permeation, which is a non-polar gas as nitrogen. The polarity of gases determines their solubility in the polymer, and permeability is directly proportional to solubility. Research results [57,58,59] show that CS increases oxygen permeation, but in this study, the obtained results (Figure 9) are in line with the results of another study, which confirms the improvement in barrier properties with an addition of CS.

In the study [60], there was a significant improvement in oxygen permeation in PVA/CS films with the increase in PVA share. In this study, it was explained that the oxygen molecules, as non-polar molecules, would dissolve to a greater extent in a less polar polymers; thus, a less polar film would have a higher oxygen permeability due to higher sorption toward oxygen. When PVA was incorporated into the CS matrix, the polarity of the matrix was weakened owing to the decrease in the amount of -NH_2_ and -OH groups. As a result, the PVA/CS films showed a decrease in oxygen barrier with the increase in PVA share.

In the study [61], oxygen permeation through PVA films decreased with the addition of CS, until the ratio of 50:50 was reached, after which with a further increase in the share of the CS, the oxygen permeation increased again. So, the lowest permeability, i.e., the best barrier properties, were observed on the PVA/CS blends of equal ratios. The reason for this can be attributed to the high effects of crosslinking caused by the intermolecular hydrogen bonds between CS and PVA molecules at that ratio.

Permeability comparison of crosslinked and non-crosslinked films shows that cross-linked films have lower permeability, i.e., crosslinking improves the barrier properties of PVA/CS blends and reduces nitrogen permeation through films. The improvement in barrier properties is probably due to the densely packed uniform distribution of GA in the structure of PVA/CS blends. In study [62], it was stated that there is a direct correlation between the permeation of oxygen molecules (non-polar as well as nitrogen) and the degree of crosslinking of the polymer film. In this research, the highest permeation of oxygen molecules was recorded in PVA film with the lowest degree of crosslinking. As the degree of crosslinking increased, the permeation of oxygen molecules through the PVA film decreased. Studies [63,64] have also shown an improvement in the barrier properties of PVA films to oxygen molecules, with the addition of GA.

**Figure 10:**
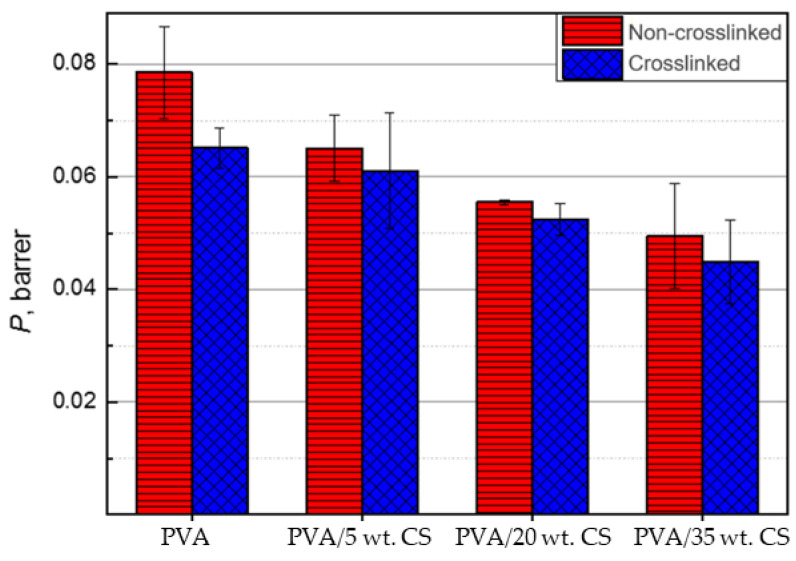
Influence of crosslinking on the permeability of PVA films with 0, 5, 20, and 35 wt. % amount of CS; data are representative of the results of repeated experiments (n = 3) and expressed as the mean value.

Based on the results shown in Figure 10, crosslinked PVA/CS films (blue) show lower permeability than non-crosslinked PVA/CS films (red), i.e., they have better barrier properties. Also, it can be clearly seen that the addition of chitosan improves the barrier properties of PVA, i.e., reduces its permeability.

## 3. Materials and Methods

### 3.1. Materials

PVA (*M_w_* 89,000–98,000, degree of deacetylation 99%), CS (*M_w_* 90,000–190,000, 75–85% deacetylated), and GA (25 wt. % aqueous solution) were purchased from Sigma Aldrich, St. Louis, MO, USA. Distilled water and 0.1 M aqueous acetic acid (Gram-Mol, Zagreb, Croatia) were used as solvents.

### 3.2. Preparation of PVA/CS Films

The films were prepared using the solvent casting method. An aqueous solution of 5 wt. % PVA was prepared by dissolving PVA powder in distilled water and heating at 80 °C with stirring for 2 h. A solution of 2.5 wt. % CS was prepared by dissolving CS in 1 wt. % acetic acid solution at room temperature with stirring for 24 h. Then the PVA and CS solutions were mixed in different ratios and stirred for 4 h to achieve homogeneous PVA/CS solutions. A half of each solution was casted into a glass mould. In the second half of each solution, 1 wt. % of GA was slowly added at room temperature and stirred for 1 h. To obtain polymer films, the resultant mixtures were poured into clean glass moulds and dried for 72 h at room temperature. After drying, two sets of polymer films with 0, 5, 20, and 35 wt. % of CS were prepared: one set of non-crosslinked polymers and one set of polymers that were crosslinked with GA. The films were carefully removed from the moulds and dried in an oven at 60 °C for 24 h, to remove the residues of water and acetic acid, and then kept in a desiccator at room temperature prior to use. The thicknesses of the obtained films were between 100 and 150 μm. The film thickness was controlled by solution mass.

### 3.3. Dynamic-Mechanical Analysis

The DMA of the polymer films was carried out using TTDMA Dynamic Mechanical Analyzer (Triton Technology, Worcester, MA, USA) in the tensile mode, at temperatures from 25 to 260 °C. The frequency was 1 Hz and the heating rate was 2 °C min^−1^. The films were cut into rectangular samples of approximately 10 × 3 × 0.15 mm. The samples were placed between two clamps and longitudinally deformed by sinusoidal stress with an amplitude of 5 μm. The values measured as a function of temperature were the storage modulus *E*′, the loss modulus *E*″, and the damping factor tan *δ* = *E*″/*E*′, where *δ* is the phase lag between stress and strain.

### 3.4. Differential Scanning Calorimetry

The thermal properties were determined by means of a DSC 823 instrument (Mettler Toledo, Columbus, OH, USA) in a nitrogen atmosphere. Two heating scans were employed: The first was carried out to eliminate the residual water and solvent in the films, and was performed at a rate of 10 °C min^−1^ from 0 °C up to 220 °C and kept at this temperature for 2 min. The second scan was carried out to determine the crystallization temperature and was performed at a heating rate of 10 °C min^−1^ from 0 °C up to 350 °C, kept at this temperature for 2 min, and then cooled down to room temperature.

### 3.5. Barrier Properties

Barrier properties were determined by the time-lag method. This is one of the simplest and most frequently used methods for the determination of permeability of polymer films. In these experiments a gas of a certain pressure comes in contact with one side of the film, and the amount of gas evolving from the opposite, low pressure, side of the film is measured as a function of time.

Permeability measurements were performed on all the crosslinked and non-crosslinked films, to examine the influence of CS nanoparticles and crosslinking on the permeation of nitrogen gas molecules through the obtained nanocomposite films. The samples were circular, with a diameter of 40 mm.

After a polymer film was placed in the measuring chamber, separating the high-pressure and low-pressure parts, the apparatus and the film were evacuated from the gases until a pressure of less than 6 × 10^−3^ mbar was reached. The evacuation was performed using a rotary vacuum pump (Pfeiffer, Kaiserslautern, Germany) for 24 h. After that, nitrogen was released into the feed side with an absolute pressure of 1.9 bar. The pressure was monitored using a digital manometer (Wika, Klingenberg am Main, Germany). Due to the difference in partial pressures between the feed and permeate sides of the apparatus, nitrogen permeated through the film. The pressure on the permeate side was monitored using a digital transmitter (Pfeiffer, Kaiserslautern, Germany) and recorded in dependence of time. All the gas permeability measurements were performed at 25 °C and at 1 bar, for 24 h.

## 4. Conclusions

Films of PVA/CS polymer blends with different amounts of CS (0, 5, 20, and 35 wt. %) crosslinked by glutaraldehyde (GA) were prepared. The addition of CS leads to a reduction in flexibility of PVA, but this can be improved with crosslinking. As the CS content increased, the viscosity of the solution and the fragility of the films increased. A small amount of CS did not significantly affect the PVA; a composition with 20 wt. % of CS preferred, while a composition with 35 wt. % of CS showed the incompatibility of the blend.

DSC analysis showed that the addition of chitosan to PVA reduces the enthalpy of fusion, while the analysis of the mechanical spectrum of DMA showed an increase in the intensity of α relaxation corresponding to the transition from the glassy to the rubbery state, due to the dynamics of molecules in the amorphous phase. CS does not have a nucleating effect, but the proportion of crystalline phase in PVA decreases and the proportion of amorphous phase increases. By reducing the degree of crystallinity, intermolecular bonds in PVA weaken, while at the same time new bonds between PVA and CS are formed. Mechanical properties are directly related to the strength of the bonds within the material. Also, the effect of CS on PVA is different depending on the temperature, with the largest increase in stiffness in the temperature range of the glass-to-rubber transition. The temperature range of the glass-to-rubber transition expands with the addition of CS, which indicates an increase in the heterogeneity of the system. The greatest effect of CS on the mechanical spectrum is in the composition with 20 wt. % of CS.

The effect of CS on PVA is also seen in nitrogen permeation, where it is expected that permeation increases with an increase in amorphous phase having a higher free volume, but stiffness of the structure due to new intermolecular bonds has the opposite effect and barrier properties are improved with CS addition. Further improvement in the barrier properties is observed after crosslinking.

PVA and CS can be chemically crosslinked with GA, where the polymers crosslink with each other and with themselves. Crosslinking reduces the mobility of molecules and increases the number of covalent bonds in the material, but at the same time it reduces the number of hydroxyl groups by branching because of the grafting of the crosslinker resulting in greater overall mobility in the material [53]. The crosslinking density depends on the amount of crosslinking agent. As a result of crosslinking, the mechanical properties at room temperature increase due to the presence of a larger number of covalent bonds, but *T_g_* decreases as a result of increased mobility. Because crosslinking takes place in solution, the effect is greater on materials that are more inclined to crystallization (0 and 5 wt. % of CS) where crosslinking prevents crystallization, but new covalent bonds increase the stiffness of the material and reduce the effect of the crystallinity decrease. The storage modulus of crosslinked PVA is 25% lower than the storage modulus of non-crosslinked PVA, both in the glassy and rubbery states. During the transition from the glassy to the rubber state, crosslinking reduces the dynamics of molecules in blends with a low CS content, while in blends with a higher CS content, the dynamics increase, indicating an additional increase in the amorphous phase. The PVA/CS blends investigated in this study have potential applications in biomedicine and pharmacy due to their non-toxicity, biocompatibility, anticancer properties, and good antimicrobial activity. Because of their low cost, simple preparation, good miscibility, and favorable physical, chemical, and biological properties, PVA/CS blends are among the most commonly used hybrid hydrogels [35,49]. Furthermore, owing to their excellent antimicrobial properties, PVA/CS blends can be also used as packaging materials in the food industry [65,66,67]. Future studies will explore the addition of ZnO nanoparticles to the PVA/CS blend to further improve its antimicrobial properties. Additionally, it is crucial to evaluate the antimicrobial properties of these systems.

## Figures and Tables

**Table 1:** Onset, maximum, and end temperatures of α relaxation and intensity of α relaxation tan *δ*_max_ for PVA films with a 0 wt. % (PVA), 5 wt. % (CS1), 20 wt. % (CS2), and 35 wt. % (CS3) of CS.

Polymer Film	α Relaxation			
	Onset	Maximum	End	Tan *δ*_max_
PVA	74.8 °C	103.8 °C	168.5 °C	0.252
PVA/CS1	75.1 °C	105.3 °C	176.0 °C	0.269
PVA/CS2	75.1 °C	114.6 °C	185.1 °C	0.305
PVA/CS3	77.1 °C	117.2 °C	189.5 °C	0.310

**Table 2:** Storage moduli *E*′ of PVA films with 0 wt. % (PVA), 5 wt. % (CS1), 20 wt. % (CS2), and 35 wt. % (CS3) of CS, at characteristic temperatures.

Material	*E*′, MPa		
	25 °C	75 °C	110 °C
PVA	6359.6	2514.0	317.4
PVA/CS1	5813.3	2395.8	319.2
PVA/CS2	6597.6	4146.2	593.7
PVA/CS3	5732.6	3118.1	476.4

**Table 3:** Onset and maximum temperatures of α relaxation for crosslinked and non-crosslinked PVA films with different amounts of CS: 0 wt. % (PVA), 5 wt. % (CS1), 20 wt. % (CS2), and 35 wt. % (CS3).

Polymer Fim	Onset Temperature of α Relaxation, °C			Maximum Temperature of α Relaxation, °C		
	Cross-Linked	Non-Crosslinked	Shift	Cross-Linked	Non-Crosslinked	Shift
PVA	52.1	74.8	22.7	100.2	105.1	4.9
PVA/CS1	49.3	75.1	25.8	98.6	108.5	9.9
PVA/CS2	69.9	75.1	5.2	105.0	114.6	9.6
PVA/CS3	73.2	77.1	3.9	112.3	116.3	4.0

**Table 4:** Intensity of α relaxation tan *δ*_max_ of crosslinked and non-crosslinked PVA films with different amounts of CS: 0 wt. % (PVA), 5 wt. % (CS1), 20 wt. % (CS2), and 35 wt. % (CS3).

Polymer Film	tan *δ*_max_		
	Crosslinked	Non-Crosslinked	Difference
PVA	0.243	0.250	2.8%
PVA/CS1	0.250	0.272	8.0%
PVA/CS2	0.324	0.305	5.8%
PVA/CS3	0.339	0.313	7.6%

## Data Availability

The data presented in this study are available upon reasonable request from the corresponding authors.
